# CD70 Deficiency due to a Novel Mutation in a Patient with Severe Chronic EBV Infection Presenting As a Periodic Fever

**DOI:** 10.3389/fimmu.2017.02015

**Published:** 2018-01-29

**Authors:** Roberta Caorsi, Marta Rusmini, Stefano Volpi, Sabrina Chiesa, Claudia Pastorino, Angela Rita Sementa, Paolo Uva, Alice Grossi, Edoardo Lanino, Maura Faraci, Francesca Minoia, Sara Signa, Paolo Picco, Alberto Martini, Isabella Ceccherini, Marco Gattorno

**Affiliations:** ^1^Clinica Pediatria e Reumatologia, Istituto Giannina Gaslini, Genova, Italy; ^2^Division of Human Genetics, Istituto Giannina Gaslini, Genova, Italy; ^3^Pathology Unit, Istituto Giannina Gaslini, Genova, Italy; ^4^Centre for Advanced Studies, Research and Development in Sardinia (CRS4), Science and Technology Park Polaris, Pula, Italy; ^5^Bone Marrow Transplantation Unit, Istituto Giannina Gaslini, Genova, Italy

**Keywords:** CD70 deficiency, periodic fever, aphthous stomatitis, pharyngitis, cervical adenitis syndrome, periodic fever, Ebstein–Barr virus, hematopoietic stem cell transplantation

## Abstract

Primary immunodeficiencies with selective susceptibility to EBV infection are rare conditions associated with severe lymphoproliferation. We followed a patient, son of consanguineous parents, referred to our center for recurrent periodic episodes of fever associated with tonsillitis and adenitis started after an infectious mononucleosis and responsive to oral steroid. An initial diagnosis of periodic fever, aphthous stomatitis, pharyngitis, cervical adenitis syndrome was done. In the following months, recurrent respiratory infections and episodes of keratitis were also observed, together with a progressive reduction of immunoglobulin levels and an increase of CD20^+^ cells. Cell sorting and EBV PCR showed 25,000 copies for 100,000 leukocytes with predominant infection of B lymphocytes. Lymph node’s biopsy revealed reactive lymphadenopathy with paracortical involvement consistent with a chronic EBV infection. Molecular analysis of *XIAP, SHA2D1A, ITK*, and *CD27* genes did not detect any pathogenic mutation. The patients underwent repeated courses of anti-CD20 therapy with only a partial control of the disease, followed by stem cell transplantation with a complete normalization of clinical and immunological features. Whole exome sequencing of the trio was performed. Among the variants identified, a novel loss of function homozygous c.163-2A>G mutation of the *CD70* gene, affecting the exon 2 AG-acceptor splice site, fit the expected recessive model of inheritance. Indeed, deficiency of both CD27, and, more recently, of its ligand CD70, has been reported as a cause of EBV-driven lymphoproliferation and hypogammaglobulinemia. Cell surface analysis of patient-derived PHA-T cell blasts and EBV-transformed lymphoblastoid cell lines confirmed absence of CD70 expression. In conclusion, we describe a case of severe chronic EBV infection caused by a novel mutation of CD70 presenting with recurrent periodic fever.

A number of different conditions are associated with the chronic activation of an EBV infection. Chronic active EBV (CABEV) was first described in 1975 (1), to describe a condition characterized by the presence of chronic symptoms of EBV infection in the absence of malignancy, autoimmunity, or a known immunodeficiency (2–4). This condition is heterogeneous from both the clinical and immunological point of view: in fact, while in some patients, EBV is nearly only detected in T or NK cells [more frequently in the Asian population (1–4)], in other patients, it is mostly detected in B cells [more common in the Caucasian population (5)].

However, during the past years, it became evident that a relevant proportion of chronic EBV infections were secondary to genetic defects leading to a selective susceptibility to EBV-induced diseases. X-linked diseases caused by mutations in *SH2D1A* (6) (XLP), *XIAP* (7), and MAGT1 (8) or autosomal recessive diseases caused by mutation in ITK (9), CORO1A (10), and FCGR3A (11) are such an example. In this condition, patients develop various degrees of lymphoproliferation and immunodeficiency, with hemophagocytic lymphohistiocytosis, hypogammaglobulinemia, and/or lymphoid malignancy secondary to chronic EBV infection as part of the clinical picture. Biallelic mutations of CD27, a tumor necrosis factor (TNF) receptor superfamily member expressed on cells of adaptive immunity and NK cells cause an EBV-associate lymphoproliferative disease with hypogammaglobulinemia (12–14). More recently, an autosomal recessive deficiency of CD70, the ligand of CD27, has been associated to a combined immunodeficiency with EBV-induced B-cell malignancy in humans (15, 16).

Though presenting with a wide heterogeneity, most of the patients with chronic EBV infection share severe clinical manifestations with early onset and poor prognosis: common immunosuppressive and antiviral therapies are usually not effective, and most of the patients not treated with bone marrow transplantation die due to lymphoid malignancies (5). Here, we describe the genetic characterization of a patient with a severe chronic EBV infection due to a novel mutation of CD70, whose initial clinical picture resembled a periodic fever syndrome.

The patient, born to consanguineous parents, presented at the age of 15 months with a not-complicated infectious mononucleosis followed by the onset of recurrent episodes of fever associated with exudative tonsillitis, adenitis, splenomegaly, and sweating, lasting 3–5 days and treated with NSAIDS or, in some occasions, with antibiotics. Blood examination revealed neutrophilic leukocytosis and elevation of acute phase reactants, while serum immunoglobulins were within the normal range. An autoinflammatory condition, consistent with periodic fever, aphthosis stomatitis, pharyngitis, cervical adenitis (PFAPA) syndrome was suspected and on-demand steroidal treatment was suggested with a prompt response. In the following months, the child continued to present periodic fever episodes with a more clear association with respiratory viral and bacterial infections and more frequent use of antibiotics. Three episodes of anterior uveitis were also observed. The patient presented several destructive dental caries (Figure 1A) and hyper sensibility to mosquitoes’ bites was reported.

**Figure 1:**
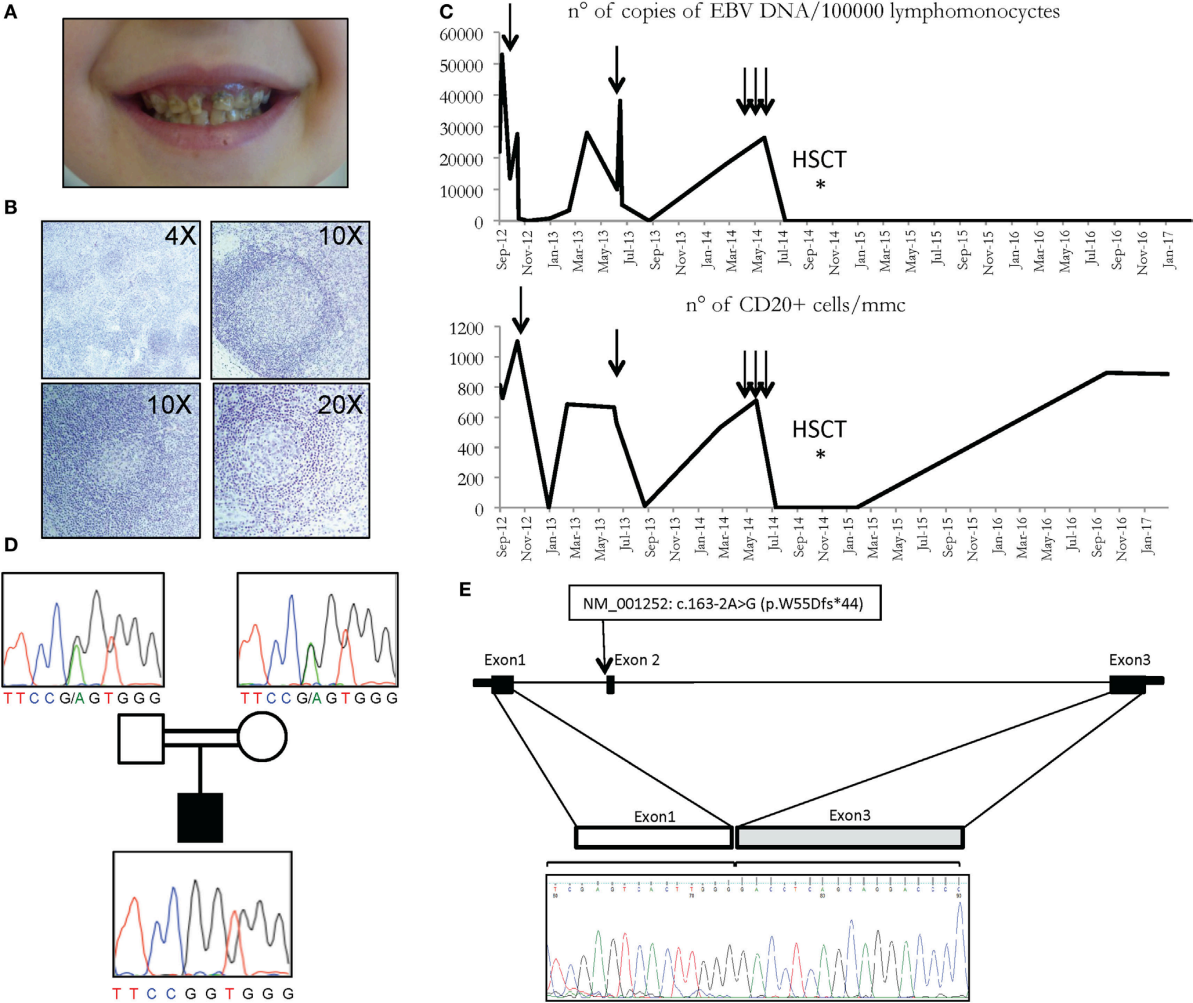
**(A)** Multiple caries in CD70 deficient patient. **(B)** Histologic analysis of patient’s lymph node showing follicular hyperplasia with paracortical expansion (top left), germinal centers with normal appearance (top right), follicular lysis (lower panels). **(C)** Copies of EBV DNA (above) and number of CD20 cells (below) detected in the patient. Arrows represent the infusions of rituximab. **(D)** Family pedigree with the c.163-2A>G variant of the *CD70* gene, heterozygous in carriers, and homozygous in the patient. **(E)** *CD70* genomic region and cDNA sequence of the first 3 exons in the patient is depicted, revealing the exon 2 skipping in the electropherogram at the bottom.

At the age of 3, immunologic tests revealed a reduction in the level of serum immunoglobulins and a reduction of both T (in particular CD3^+^CD8^+^ cells) and B lymphocytes populations (Table 1). Quantitative PCR for EBV DNA revealed 21,935 copies for 100,000 leukocytes with prevalence of infection in the B cells (Table 2). Whole body positron emission tomography revealed a retroperitoneal formation of about 35 mm with an increased metabolism. At biopsy, staining was compatible with reactive lymphadenopathy with paracortical involvement associated with EBV infection (Figure 1B). No signs of lymphoma were observed. The cytofluorimetric characterization of the immunophenotype enlightened the presence of a mixed lymphocyte population composed of polyclonal T and B lymphocytes.

**Table 1:** lymphocytes population detected in the patient.

Lymphocytes’ population	Percentage of lymphocytes (absolute count/mmc)
CD3+	76 % (4071)
CD3+ CD4+	42.5 % (2246)
CD3+ CD8+	26.2 % (134)
CD19+	15.4 % (814)
CD20+	15 % (800)
CD3– CD16+ CD56+	7 % (370)

**Table 2:** EBV DNA quantitative PCR in different lymphocytes populations.

Lymphocytes’ population	EBV DNA quantitative PCR	% of infected cells
CD3+ CD8+	8100 copies/ 1812200 cells	0.4 %
CD3+ CD4+	10700 copies/ 4584300 cells	0.2 %
CD3– CD16+ CD56+	1700 copies/ 367000 cells	0.5 %
CD19+	386540 copies/ 4500000 cells	8.6 %

Most common genetic conditions possibly associated with chronic EBV infection and hypogammaglobulinemia were ruled out by molecular analysis of the coding sequence of target genes (*SHA2D1A, XIAP, BAFF-R*, and *ICOS*). In addition, the cytofluorimetric analysis of perforin, CD107, and 2B4 receptor was normal (data not shown). In light of these findings, a severe chronic EBV infection was suspected.

Taking into consideration, the overall satisfactory general conditions and in light of the prevalent involvement of CD20^+^ lymphocytes, after informed consent approved by G. Gaslini ethical board, treatment with rituximab (375 mg/m^2^/dose) was started with a good clinical response and a dramatic reduction of viral load (Figure 1C). The patient was followed longitudinally with the indication to repeat rituximab whenever the viral load exceeded 20,000 copies/100,000 lymphomonocytic cells and/or the re-appearance of fever and other manifestations associated with EBV. During the following 2 years, the patient received only two administrations of rituximab, in association with i.v. immunoglobulin substitutive treatment every 6 weeks. The patient presented a general wellbeing with a regular growth, without severe infections, and with persistent control of the viral load and of the number of CD20 cells (Figure 1C). However, 3 years after the diagnosis a clear progression of the disease was observed, with subsequent elevations of the viral load and an increase in the frequency and severity of respiratory tract infections requiring, in some cases, prolonged hospitalization. In this period, the patient received three consecutive administrations of rituximab, with only a transient clinical response. On these bases, hematopoietic stem cell transplantation (HSCT) was planned.

The conditioning regimen consisted of Thiotepa (8 mg/kg, day −7), Fludarabine (40 mg/m^2^/day, from day −6 to day −3), and Treosulfan (14 gr/m^2^ from day −6 to day −4). Rabbit anti-thymocyte globulin (ATG, 30 mg/kg from day −4 to −2), micophenolyc acid (30 mg/kg from day 0), and cyclosporine (1 mg/kg until day −2 and 3 mg/kg from day −1) were administered as Graft-versus-Host Disease (GvHD) prophylaxis, whereas a single dose of rituximab (200 mg/sqm) was administered on day 0 for prevention of EBV-related lymphoprolipherative disease.

A total of 2.88 × 10^8^/kg mononuclear bone marrow cells were infused from an 8/10, EBV positive HLA-matched unrelated female donor (1 antigenic HLA-C mismatch in host-versus-graft direction, 1 bidirectional HLA-DQB1 mismatch). Neutrophils and platelets engraftment occurred on day +24 and +18 after HSCT, respectively. At first evaluation, chimerism analysis revealed a mixed pattern (88% donor cell), shown to be of full donor origin, which persisted at all subsequent evaluations. The early post-transplant phase was complicated by mild mucositis, multiple CMV reactivations successfully treated by Foscarnet and/or Gancyclovir, grade 2 acute GvHD (grade 2) responsive to corticosteroids. Immunosuppression was progressively tapered and eventually discontinued 16 months after HSCT in the absence of any manifestation of chronic GvHD. Blood EBV DNA never positivized after HSCT, and specific antibodies were detected since month +9. After 2 years and 6 months from HSCT, the patient persists in good health in the absence of any sign of the disease.

Having excluded some of the most common genetic causes of genetic susceptibility to EBV infections, and in light of the severity of the clinical picture, and the consanguinity of the parents (Figure 1D), a whole exome sequencing (WES) approach was undertaken in the patients and their parents. Variants were prioritized with a custom pipeline to identify the genetic cause of patient’s condition. In particular, only variants either unreported or already reported in the general population with a frequency lower than 1% were considered. Moreover, synonymous, intronic, and UTR variants were excluded, in addition to splicing variants not specifically affecting the donor and acceptor splice sites. Among the homozygous variants, thus identified (Table S1 in Supplementary Material), a splicing variant of the CD70 gene, fitting the expected recessive model of inheritance, with the parents being heterozygous for the same mutation (Figure 1D), was further investigated. In particular, the variant c.163-2A>G affects the exon 2AG-acceptor splice site of the *CD70* gene (NM_001252). To analyze the effect of the mutation on the transcription of the gene, we sequenced the complementary DNA, revealing the skipping of exon 2 (Figure 1E).

Flow cytometry analysis of CD70 expression of patient’s PHA-T cell blasts at 7 and 23 days of stimulation failed to detect even low amounts of CD70 protein. In contrast, expression of CD70 was detected on a fraction of PHA-stimulated T cells from healthy donors (Figure 2A). Similarly, CD70 was not detected on EBV-trasformed lymphoblastoid cells lines (EBV-LCL) derived from the patient, in contrast to EBV-LCL from healthy donors that expressed high levels of CD70 on their surface (Figure 2B). After HSCT, the expression of CD70 in patient’s PHA-T cell blasts at 12 days of culture was comparable to healthy donor (Figure 2C). These data demonstrate that c.163-2A>G mutation causes exon skipping and absence of CD70 protein expression in patient’s cells.

**Figure 2:**
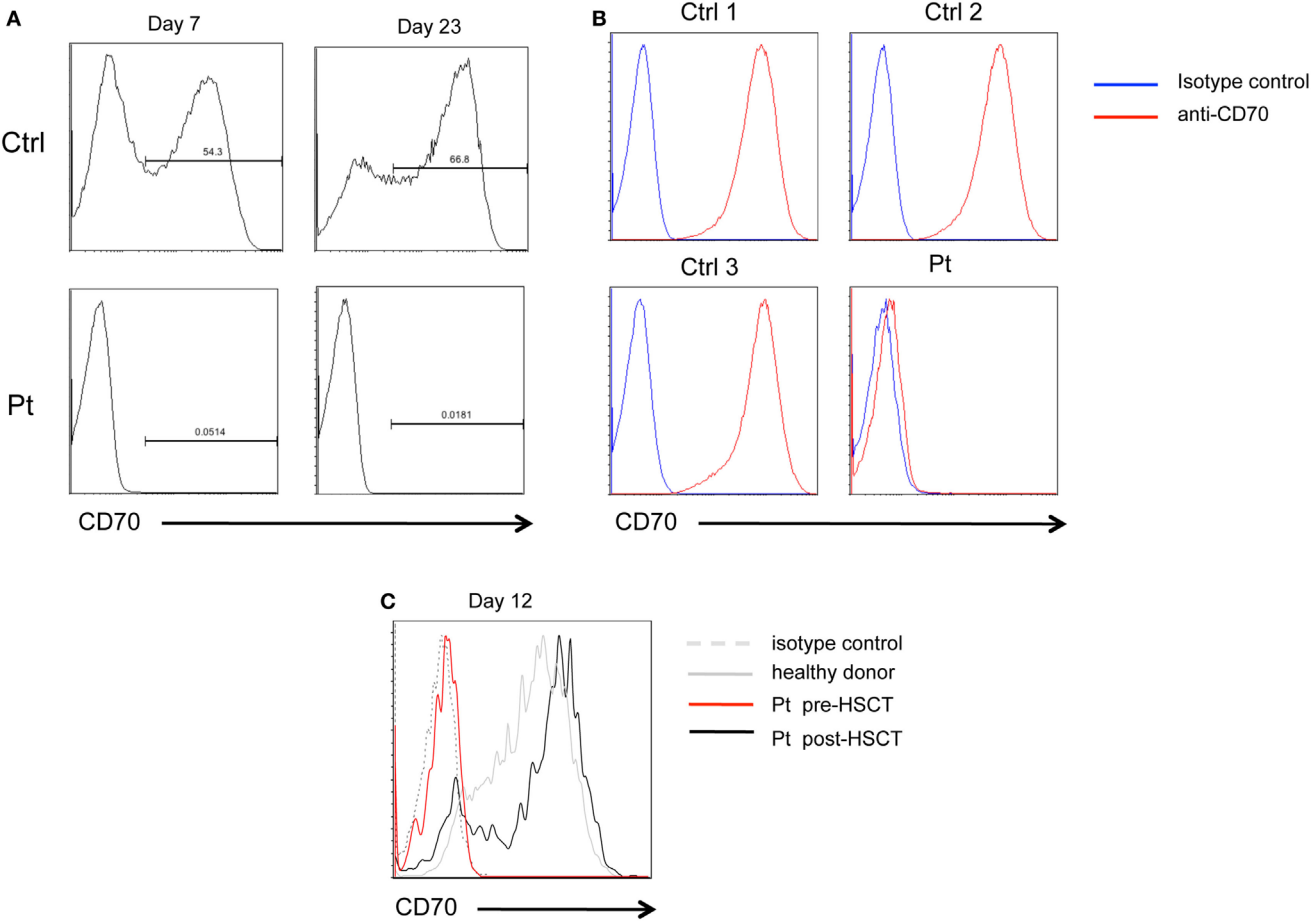
CD70 expression. **(A)** FACS histograms showing CD70 expression detected with anti-CD70 antibody on PHA-stimulated CD3^+^ T cell blasts from patient (Pt) or healthy donor (Ctrl), at day 7 and 23 of culture. **(B)** FACS histograms showing CD70 expression on lymphoblastoid cell lines derived from the Pt or three healthy donors (Ctrl1, Ctrl2, and Ctrl3) detected with anti-CD70 antibody. **(C)** FACS histograms showing CD70 expression on PHA-stimulated CD3^+^CD4^+^ T cell blasts from Pt pre-HSCT (red line), Pt post-HSCT (black line), or healthy donor (dashed gray line), at day 12 of culture.

CD70 is the ligand of the TNF superfamily member CD27 and is expressed by antigen-presenting cells upon triggering of CD40 and toll-like receptors (TLR), by T cells upon TCR activation, CD28 cross-linking, and cytokine exposure, and constitutively by thymic epithelial cells (17). CD27–CD70 binding provides a costimulatory signal for CD4 and CD8 activation (18, 19), and studies in mice have provided evidences for its role in memory expansion and protection upon reinfection (20, 21). Inborn errors of *CD27* are a well-known cause of persistent symptomatic EBV viremia and hypogammaglobulinemia, thus making *CD70* mutations the likely cause of our patient’s phenotype.

While our characterization of this novel immune defect was on-going, two groups reported the association of *CD70* mutations with combined immunodeficiency in a total of five patients affected by EBV-associated Hodking’s lymphoma and hypogammaglobulinemia (15, 16). The groups demonstrated that CD70 deficiency (16) causes a reduction of *in vitro*-generated EBV-specific cytotoxic T cell activity and to a decreased expression of 2B4 and NKG2D, receptors implicated in controlling EBV infection, on memory CD8^+^ T cells, consistent with their impaired capability to kill EBV-infected cells (15).

Four of the five reported patients suffered by EBV-associated Hodgkin’s lymphoma with an onset at 2.5, 3 (two patients), and 17 years of age and hypogammaglobulinemia. The initial presentation was a severe varicella infection with pneumonia in one case, encephalitis of unknown cause in another one, and HL in the other three cases. Our patient uniquely presented with a subtle history of recurrent fever and inflammatory symptoms was similar to PFAPA syndrome. Of note, the patient reported by Izawa et al. following treatment for HL, also presented with recurrent fever and lymphoadenopathy, while P1 in Abolhassani et al. presented oral aphthous ulcers.

In conclusion, we report an early diagnosed case of CD70 deficiency with an onset of periodic fever, suggesting that in EBV positive patients with signs of PFAPA syndrome molecular analysis of *CD70* gene should be performed.

## Materials and Methods

### Informed Consent

Experiments and molecular genetic analysis were performed, following informed consent and approval by the institute review board. The family gave permission for publication of clinical and laboratory data and photographic images.

### Cell Culture

Whole blood samples were collected from the patient and healthy donors. Peripheral blood mononuclear cells (PBMC) were isolated by Ficoll-Paque density gradient from blood samples using standard procedures. Expansion of T cell blasts were obtained by incubating PBMCs for 48 h with 1 µg/ml of phytohemagglutinin (PHA) (Sigma-Aldrich) in RPMI supplemented with 10% FBS serum, 1% penicillin, and 1% streptomycin. After 2 days, PHA-blasts were maintained in culture with 100 U/ml IL-2.

EBV-trasformed cell lines were generated from the patient and control healthy donors with standard technique.

### Lymph Node Histology

Lymph nodes from diagnostic biopsies were fixed in 4% formalin, paraffin embedded and sectioned for H&E staining with standard techniques. Images were recorded using a Zeiss Axioskop plus microscope mounting a digital microscopy camera AxioCam ICc5.

### Flow Cytometry

Cell staining and phenotype analyses of blast T cells and cell lines were performed according to standard flow cytometry methods.

Expression of CD70 on PHA-T cell blasts pre- and post-HSCT and lymphoblastoid cell lines derived from the patient and four healthy donors was evaluated by flow cytometry. Anti-CD70 PE monoclonal antibody (clone: Ki-24, isotype: mouse IgG3, k, BD Biosciences), PeCy7 mouse anti-human CD3 (clone: SK7, isotype: mouse BALB/c IgG1, k, BD Biosciences), APC mouse anti-human CD4 (clone: RPA-T4, isotype: mouse IgG, k, BD Biosciences), and PC7 mouse anti-human CD19 (clone: J3-119, isotype: IgG1, mouse, Beckman Coulter) were used.

### WES and Sanger Sequencing Validation

Molecular analysis of *SHA2D1A, XIAP, BAFF-R*, and *ICOS* genes was performed through Sanger sequencing in a CLIA certified laboratory.

Whole exome sequencing consisted of several steps. Exome capture was performed on genomic DNA using the Nextera Rapid Capture Expanded Exome Kit (Illumina Inc., San Diego, CA, USA) according to manufacturer instructions. The enriched libraries were sequenced on the Hiseq3000 instrument with 100 bp paired-end reads. This approach achieved an 86× average coverage over the 62Mb of target regions sequenced, with more than 95% regions covered. Data analysis has been performed using an analysis pipeline implemented in Orione (22). Briefly, paired-end sequence reads were aligned to the human genome (hg19) with BWA-MEM [v.0.7.9a (23)]. Initial mappings were processed using the GATK framework [version 2.8.1 (24)], according to the GATK best practices recommendations (25, 26). Variants were classified as known or novel based on dbSNP146 and annotated using KGGSeq (27).

Annotations included positions in UCSC, RefGene, GENCODE and ENSEMBL transcripts, OMIM and ClinVar annotations, potential false positive signals, allele frequency in dbSNP, ESP6500, 1000 Genome Project (release 05/2013), and ExAC, functional predictions for the amino acid changes according to different models (SIFT, Polyphen2, LRT, MutationTaster, MutationAssessor, and FATHMM) retrieved from dbNSFP v 2.9 (database of human nonsynonymous SNPs and their functional predictions) (28).

Homozygous missense and splicing mutations at ±2bp were taken into account and selected based on their allele frequency (variants unreported or reported with a frequency of <1% in the general population were selected). The identified CD70 variant was validated by Sanger sequencing both in the proband and in his parents. PCR products were purified by ExoSAP-IT (GE Healthcare) and directly sequenced using Big Dye v1.1 and an ABI3130 automated sequencer (Applied Biosystems, Foster City, CA, USA).

### CD70 Transcript Characterization

Total RNA from patient’s T cells and from three-unrelated controls was isolated by a commercial RNA purification kit (RNeasy Mini kit, Qiagen, GmbH, Germany) and 1 µg of total RNA was reverse transcribed by iScript cDNA synthesis kit (Bio-Rad Laboratories) according to the manufacturer’s protocol. DNA amplification followed by Sanger sequencing was carried out on the cDNA, thus obtained using CD70 specific primers designed on exon 1 (5′- GTGATCTGCCTCGTGGTGT-3′) and exon 3 (5′-AGGCAATGGTACAACCTTGG-3′).

## Ethics Statement

All subjects gave written informed consent in accordance with the Declaration of Helsinki. The protocol was approved by the Regional Ethical Committee.

## Author Contributions

RC, EL, MF, FM, SS, PP, AM, and MG clinically followed the patient. MR, PU, AG and IC performed genetic analysis. ARS performed pathological studies. SC and CP performed *in vitro* studies. RC, MR, and SV wrote the manuscript and prepared the figures. SC contributed to the writing and prepared the figures. IC and MG supervised all experiments and edited the manuscript.

## Conflict of Interest Statement

The authors declare that the research was conducted in the absence of any commercial or financial relationships that could be construed as a potential conflict of interest.
